# Supplementary material for: Transmissibility of coronavirus disease 2019 in Chinese cities with different dynamics of imported cases
Source: PeerJ. 2020 Nov 6;8:e10350. doi: 10.7717/peerj.10350 (PMC7651459; doi:10.7717/peerj.10350)
Supplement: Supplemental Information 1 [file peerj-08-10350-s001.pdf]

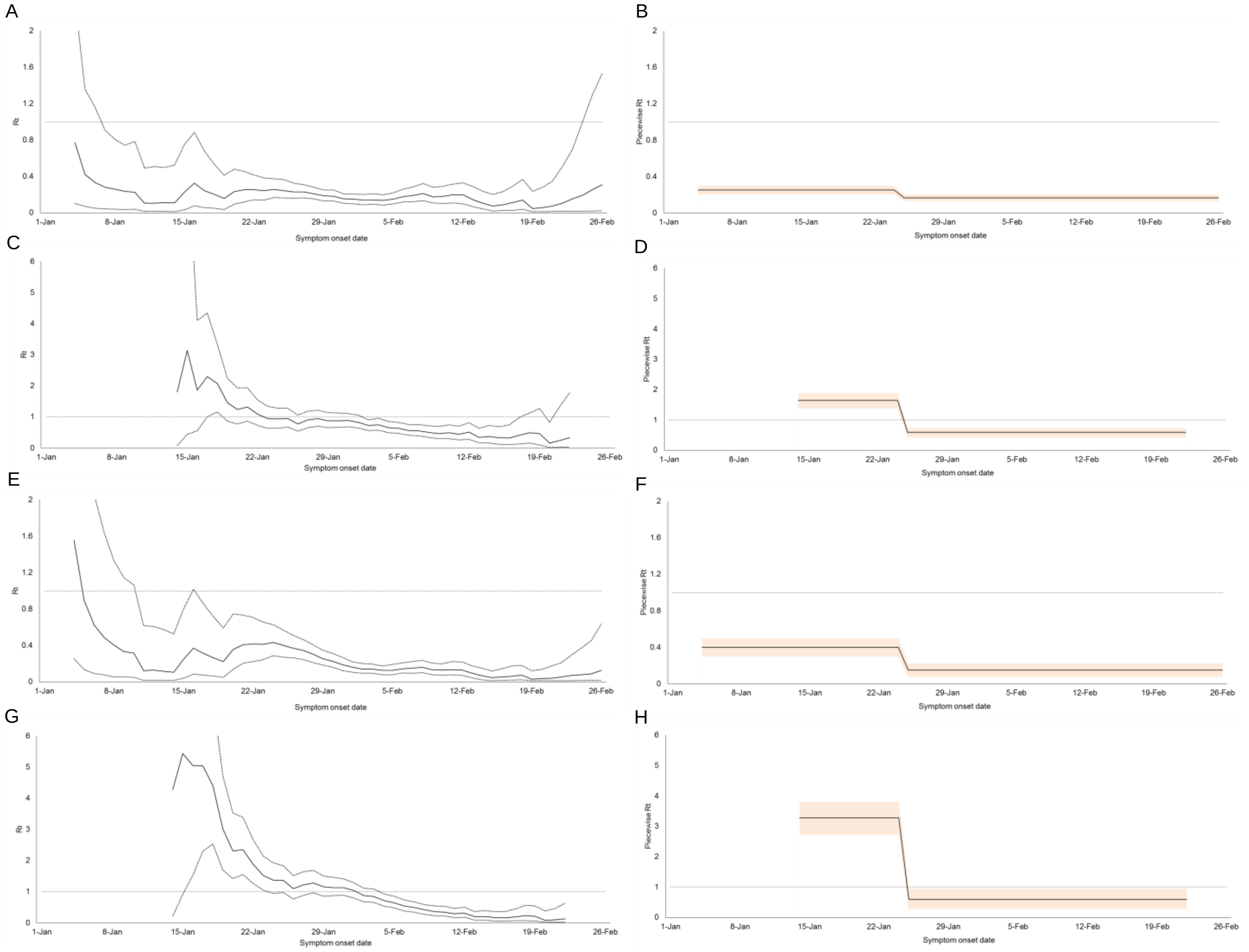

Figure S1. Time-varying reproduction number (R_t) (left panel) and piecewise R_t (right panel) assuming a shorter (A-D) and longer serial interval (E-H) in Shenzhen (A, B, E, and F) and Hangzhou (C, D, G, and H) respectively.

Table S1. Number of local and imported cases by illness onset date in Hangzhou, China

Illness onset date	Number of local cases	Number of Imported cases
01/01/2020	0	0
01/02/2020	0	0
01/03/2020	0	0
01/04/2020	0	0
01/05/2020	0	0
01/06/2020	0	0
01/07/2020	0	0
01/08/2020	0	0
01/09/2020	0	0
01/10/2020	0	0
01/11/2020	0	0
01/12/2020	0	0
01/13/2020	0	1
01/14/2020	0	0
01/15/2020	1	2
01/16/2020	2	0
01/17/2020	4	2
01/18/2020	6	0
01/19/2020	2	1
01/20/2020	2	4
01/21/2020	7	2
01/22/2020	2	2
01/23/2020	2	1
01/24/2020	4	5
01/25/2020	9	3
01/26/2020	4	6
01/27/2020	11	3
01/28/2020	11	2
01/29/2020	9	4
01/30/2020	7	3
01/31/2020	8	1
02/01/2020	5	3
02/02/2020	3	1
02/03/2020	5	2
02/04/2020	3	0
02/05/2020	6	0
02/06/2020	1	0
02/07/2020	2	0
02/08/2020	1	0
02/09/2020	0	0
02/10/2020	0	1
02/11/2020	1	0
02/12/2020	2	0
02/13/2020	0	0
02/14/2020	0	0
02/15/2020	0	0
02/16/2020	0	0
02/17/2020	0	0
02/18/2020	0	0
02/19/2020	0	0
02/20/2020	0	0
02/21/2020	0	0
02/22/2020	0	0
02/23/2020	0	0
02/24/2020	0	0
02/25/2020	0	0
02/26/2020	0	0

Table S2. Number of local and imported cases by illness onset date in Shenzhen, China

Illness onset date	Number of local cases	Number of Imported cases
01/01/2020	0	2
01/02/2020	0	0
01/03/2020	1	1
01/04/2020	0	3
01/05/2020	0	0
01/06/2020	0	1
01/07/2020	0	0
01/08/2020	0	1
01/09/2020	0	1
01/10/2020	0	0
01/11/2020	0	1
01/12/2020	0	3
01/13/2020	0	1
01/14/2020	0	0
01/15/2020	1	1
01/16/2020	1	6
01/17/2020	0	4
01/18/2020	0	7
01/19/2020	0	13
01/20/2020	3	16
01/21/2020	3	13
01/22/2020	3	25
01/23/2020	5	30
01/24/2020	8	32
01/25/2020	5	20
01/26/2020	4	27
01/27/2020	5	22
01/28/2020	4	15
01/29/2020	1	13
01/30/2020	3	15
01/31/2020	0	7
02/01/2020	5	18
02/02/2020	2	6
02/03/2020	3	7
02/04/2020	2	6
02/05/2020	4	8
02/06/2020	3	3
02/07/2020	2	1
02/08/2020	0	6
02/09/2020	0	3
02/10/2020	0	0
02/11/2020	2	0
02/12/2020	0	4
02/13/2020	0	2
02/14/2020	0	2
02/15/2020	0	0
02/16/2020	0	0
02/17/2020	0	0
02/18/2020	0	0
02/19/2020	0	0
02/20/2020	0	0
02/21/2020	0	0
02/22/2020	0	0
02/23/2020	0	1
02/24/2020	0	0
02/25/2020	0	0
02/26/2020	0	0
